# Expression Profile Analysis of the Cell Cycle in Diploid and Tetraploid *Carassius auratus* red var.

**DOI:** 10.3389/fgene.2020.00203

**Published:** 2020-03-17

**Authors:** Li Ren, Jiahao Lu, Yunpeng Fan, Yibo Hu, Jiaming Li, Yamei Xiao, Shaojun Liu

**Affiliations:** ^1^State Key Laboratory of Developmental Biology of Freshwater Fish, Hunan Normal University, Changsha, China; ^2^College of Life Sciences, Hunan Normal University, Changsha, China

**Keywords:** polyploidy, *in vitro*, *in vivo*, cell cycle, mRNA expression

## Abstract

Polyploidization often leads to “transcriptome shock,” and is considered an important factor in evolution of species. Analysis of the cell cycle, which is associated with survival in polyploidy, has proved useful in investigating polyploidization. Here, we used mRNA sequencing to investigate global expression *in vitro* (in cultured cells) and *in vivo* (in fin and liver tissues) in both the diploid and tetraploid *Carassius auratus* red var.. Differential expression (DE) of genes in diploid (7482, 36.0%) and tetraploid (3787, 18.2%) states suggested that *in vitro* and *in vivo* conditions dramatically change mRNA expression levels. However, of the 20,771 total shared expressed genes, 18,050 (87.0%), including 17,905 (86.2%) non-differentially expressed genes (DEGs) and 145 (0.7%) DEGs between diploids and tetraploids, showed the same expression trends in both cultured cells and liver tissues. Of the DEGs, four of seven genes in the cell cycle pathway had the same expression trends (upregulated in diploids and tetraploids) in both cultured cells and liver tissues. Quantitative PCR analysis confirmed the same expression trends in the nine DEGs associated with regulation of the cell cycle. This research on common characteristics between diploids and tetraploids provides insights into the potential molecular regulatory mechanisms of polyploidization. The steady changes that occur between diploids and tetraploids *in vitro* and *in vivo* show the potential value of studying polyploidy processes using cultured cell lines, especially with respect to cell cycle regulation.

## Introduction

Polyploidy occurs in plants, animals, and fungi (Comai, 2005; Blomme et al., 2006). It plays an important role in the evolutionary history of species by providing a large amount of genetic material, contributing to the genomic complexity, and further promoting speciation (Comai, 2005; Blomme et al., 2006; Otto, 2007). Polyploid breeding induced by artificial and natural mutagenesis is utilized to obtain cells and organisms with genome duplication, contributing to obtaining polyploid animals to achieve high genome plasticity, including allotetraploid hybrids of *Carassius auratus* red var. and *Cyprinus carpio* L. (Liu et al., 2001, 2016), polyploid channel catfish (*Ictalurus punctatus*) (Goudie et al., 1995), polyploid shellfish (Francesc et al., 2009), and autotetraploid *C. auratus* red var. × *Megalobrama amblycephala* (Qin et al., 2014).

Besides polyploid individuals, polyploidy has also been found in cells and tissues of diploid organisms, such as human muscle tissues, megakaryocytes, and hepatocytes (Parmacek and Epstein, 2009), as well as in some tissues under conditions of stress, such as aging seminal vesicle cells (Nguyen and Ravid, 2010). Additionally, polyploidy was shown to occur after administration of the drug cisplatin (Cantero et al., 2006) and the c-Jun N-terminal kinase inhibitor SP600123 (Zhou et al., 2016). Genetic instability in polyploid cells might lead to aneuploidy, thereby contributing to the formation of cancer (Storchova and Pellman, 2004). However, after self-breeding the allotetraploid progeny of *C. auratus* red var. and *C. carpio* L. for 26 generations, analysis of the chromosome number and reproductive fertility had revealed its genetic stability (Liu et al., 2001, 2016). To further study polyploid fish, the establishment of *in vitro* cell culture is necessary to analyze complex regulatory mechanisms including genome-wide additive and dominant expression in polyploid formation (Yoo et al., 2013).

Fibroblasts are the main cellular components of connective tissue, and can be easily obtained and cultured *in vitro*; they have been widely used to study the senescence of cells, cell damage, some congenital metabolic abnormalities and enzyme defects in basic medicine and clinical medicine research (Shima et al., 1980; Shima, 1988; Mahale et al., 2008; Swaminathan et al., 2016). Previously, cultured fibroblasts were obtained from the tail fin tissue of *C. auratus* red var. and their allotetraploid offspring (Huang et al., 2017). Here, we present an analysis of mRNA expression to investigate the cultured cells and tissues of diploid and tetraploid *C. auratus* red var.. We performed differential expression (DE) analysis between diploid and tetraploid samples in cultured fibroblasts and liver tissues. We also identified a number of mRNAs of differentially expressed genes (DEGs), and used quantitative (q) PCR to further confirm our findings in cultured cells and fin and liver tissues. Analysis of global expression in cultured cells and tissues should help to reveal whether *in vitro* cell lines can be used to research molecular expression and regulatory mechanisms in polyploid fish.

## Materials and Methods

### Sample Preparation

All experiments were approved by the Animal Care Committee of Hunan Normal University and followed guidelines of the Administration of Affairs Concerning Animal Experimentation of China. *C. auratus* red var. was distributed in natural waters of China, and tetraploid *C. auratus* red var. × *C. carpio* L. were obtained from self-crossing of the allodiploid hybrid F_2_ of *C. auratus* red var. (♀) × *C. carpio* L. (♂) (Liu et al., 2001, 2016). These individuals were bred and fed in pools under the same water temperature, dissolved oxygen content, and foraging conditions at the Engineering Research Center of Polyploid Fish Breeding and Reproduction of the State Education Ministry, China. Three individuals of each species were collected for further study.

Diploid cultured cells were obtained from the caudal fin of *C. auratus* red var., and tetraploid cultured cells were derived from the caudal fin of a tetraploid hybrid of *C. auratus* red var. (♀) × *C. carpio* L. (♂). Cells were cultured in complete growth medium composed of Dulbecco’s modified Eagle’s medium (Sigma) supplemented with 100 U/ml penicillin, 100 μg/ml streptomycin (Invitrogen, Carlsbad, CA, United States), 10% fetal bovine serum (Invitrogen, Carlsbad, CA, United States), 0.1% 2-mercaptoethanol (Invitrogen, Carlsbad, CA, United States), 1 mM sodium pyruvate (Invitrogen, Carlsbad, CA, United States), and 1 mM non-essential amino acids (Invitrogen, Carlsbad, CA, United States). Cells were grown in 5% (v/v) CO_2_ at 28°C.

### Determination of Ploidy Level

Before extracting total RNA, the ploidy level and DNA content of each sample were confirmed by flow cytometry. Diploid *C. auratus* red var. was used as a control group. Fish were anesthetized with 100 mg/L MS-222 (Sigma) before dissection. Fish tissues (∼0.2 cm^2^) were quickly rinsed with 70% alcohol and washed with phosphate-buffered saline. They were then digested with 0.25% trypsin (Invitrogen, Carlsbad, CA, United States) for 15–30 min.

### RNA Extraction

Total RNA was extracted from cultured cells, fin and liver tissues in accordance with a standard TRIzol protocol (Invitrogen, Carlsbad, CA, United States) after RNALater removal (Hummon et al., 2007). The purified RNA was quantified using a 2100 Bioanalyzer system (Agilent). Then, the RNA was used to obtain first-strand cDNA synthesized using AMV reverse transcriptase (Fermentas), with an oligo (dT)_12__–__18_ primer at 42°C for 60 min and 70°C for 5 min.

### Obtaining Transcriptome Data

For this study, we focused on the transcriptional regulation of *C. auratus* red var. *in vitro* and *in vivo* to investigate whether there is a difference in cell cycle regulation. Therefore, we obtained mRNA sequencing (seq) data of the liver tissue of diploid *C. auratus* red var. and tetraploid *C. auratus* red var. (♀) × *C. carpio* L. (♂) from the NCBI SRA database (SRR538839, SRR542431, SRR1535135, and SRR1536195) (Liu et al., 2016). Next, we submitted the mRNA-seq data of *in vitro* diploid *C. auratus* red var. and tetraploid *C. auratus* red var. (♀) × *C. carpio* L. cultured cells to the NCBI SRA (SRR7640867, SRR7640866, SRR7640869, and SRR7640868).

### Mapping and Differential Expression Analysis

After removing read adapters and low-quality reads, quality of all clean reads of each library was assessed using the FastQC program^1^. Principal component analysis was used to examine the contribution of each gene to the separation of classes in the six liver transcriptomes based on Euclidean distances (Anders and Huber, 2010). mRNA-seq reads from each sample were mapped against the reference genome (*C. auratus* red var.^2^) using TopHat with default parameters (Trapnell et al., 2012). Negative effects of background noise were removed based on the read counts (≤2) of genes in all biological replicates. To compare DE between diploid and tetraploid *C. auratus in vitro* and *in vivo*, the values of fragments per kilobase of transcript per million mapped reads (Mortazavi et al., 2008) were calculated using Cufflinks (version 2.1.0) (Trapnell et al., 2012). The false discovery rate (FDR) was used to determine the threshold *P-*value in multiple tests and analysis. Genes with FDR ≤ 0.01 and fold change (FC) > 2 were defined as the DE threshold using the DEGseq package of the R program (version 2.13) (R Foundation for Statistical Computing, Vienna, Austria) (Wang et al., 2010). DEGs were annotated using Gene Ontology (GO) and Kyoto Encyclopedia of Genes and Genomes (KEGG) databases.

### Determination of DEGs Using Quantitative RCR

Quantitative (q) PCR primers to amplify 11 cell-cycle-regulated genes (*lc3*, *smad6*, *p53*, *myc*, *gng10*, *id1*, *gng12*, *gadd45*, *jun*, *calm*, and *erg1*) were designed using conserved regions of coding sequences in the reference genome (Supplementary Table 1). Primers were used to detect expression with the ABI Prism 7500 Sequence Detection System (Applied Biosystems) and the following amplification conditions: 50°C for 5 min then 95°C for 10 min, followed by 36 cycles of 95°C for 15 s and 60°C for 45 s. Each test was performed three times. Relative quantification was performed and melting curve analysis was used to verify the generation of a single product at the end of the assay. Triplicates of each sample were used both for standard curve generation and during experimental assays. The relative expression of each gene was calibrated with β-actin, and relative mRNA expression data were analyzed using the 2^–Δ^ ^Δ^ ^*Ct*^ method (Livak and Schmittgen, 2001). The expression level of β-actin in induced tetraploid *C. auratus* red var. was estimated by the ratio of transcript abundance to the gene copy number using PCR and qPCR of DNA and RNA, respectively, extracted from cultured cells, caudal fin tissues, and liver tissues in diploid and tetraploid states. β-Actin expression was compared between diploid and tetraploid states.

## Results

### Expression Patterns in Diploids and Tetraploids

**FIGURE 1:**
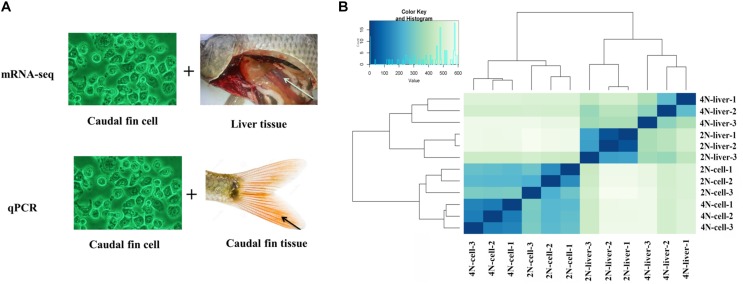
Strategy of the expression analysis and expression cluster in all samples. **(A)** mRNA-seq and qPCR methods were used to determine the expression levels in cultured cells, caudal fin tissues, and liver tissues. The comparison of “vs. 1” and “vs. 2” was used to assess the DE of *in vivo* and *in vitro* between diploids and tetraploids. The comparison of “vs. 3” and “vs. 4” was used to assess the DE of diploids and tetraploids between *in vivo* and *in vitro*. **(B)** Overall clustering of 12 samples including diploid and tetraploid liver tissues, and diploid (2N) and tetraploid (4N) cultured cells, using normalized count data calculated by Cufflinks. The heatmap drawn from all gene count data for the reference genome depicts the relationships of all transcriptomes.

To examine changes in the global transcriptomic profile between diploid and tetraploid *C. auratus* red var. *in vitro* and *in vivo*, 12 transcriptomes (from liver tissues and cultured cells; three individuals each from diploid and tetraploid) were obtained by paired-end sequencing. After initial adapter trimming and quality control, 535.9 million cleaned reads from the 12 libraries were obtained (Supplementary Table 2). Among these, 451.7 million cleaned reads were mapped against the reference genome of *C. auratus* red var.^3^ using TopHat (Supplementary Table 2). The heatmap based on Euclidean distances clustered the diploid liver and tetraploid cultured cell samples. These results indicated significant differences in expression between liver tissues and cultured cells (Figure 1). The analysis of expression levels between diploids and tetraploids revealed the presence of silent transcripts based on a threshold of >10 reads for each gene (Ren et al., 2016). Four shared silent genes were detected in both diploid cultured cells and diploid liver samples, while only one shared gene was found in both of the tetraploid samples (Supplementary Figure 1).

### DE Analysis *in vitro* and *in vivo* Using mRNA-Seq

After obtaining mapping information for all transcriptomes, we identified 20,771 shared expressed genes. To compare DE between *in vitro* and *in vivo* conditions, we performed DE analysis of diploid cultured cells and liver tissues (vs. 3 in Figure 1A) for all 20,771 expressed genes. A total of 3603 (17.3%) genes were found to be upregulated in diploid cultured cells, while 3879 (18.7%) were upregulated in diploid liver samples (Supplementary Figure 2). GO analysis of categories with the largest numbers of DEGs showed that 620 DEGs belonged to cell part (GO: 0044464) in the main category of cellular component, 1149 belonged to binding (GO: 0005488) in the main category of molecular function, and 1013 belonged to cellular process (GO: 0009987) in the main category of biological process (Supplementary Figure 3).

**FIGURE 2:**
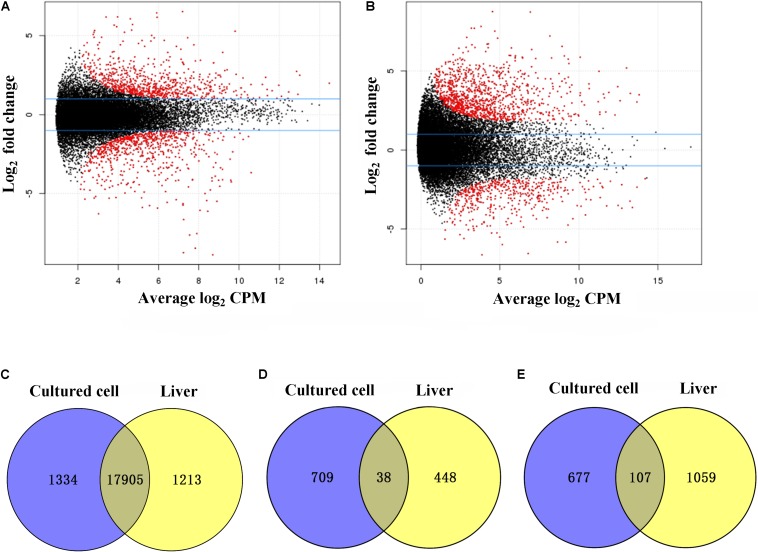
Differentially expressed genes (DEGs) between diploid and tetraploid states in cultured cells and liver tissues. **(A)** The distribution of DEGs in cultured cells. **(B)** The distribution of DEGs in liver tissues. **(C)** Shared genes with no DE in cultured cell and liver samples. Log_2_ counts per million (CPM). **(D)** Shared upregulated genes in diploid cultured cells and liver samples. **(E)** Shared upregulated genes in tetraploid cultured cells and liver samples.

Next, we focused on differences in expression between tetraploid cultured cells and liver tissues (vs. 4 in Figure 1A), and identified 3787 (18.2%) DEGs (Supplementary Figure 2). Among these, 1258 were upregulated in cultured cells and 2529 were upregulated in the liver (Figure 2B). GO analysis of categories with the largest numbers of genes showed that 195 DEGs belonged to cell part (GO: 0044464) in the main category of cellular component, 557 belonged to binding (GO: 0005488) in the main category of molecular function, and 392 belonged to cellular process (GO: 0009987) in the main category of biological process (Supplementary Figure 3).

### DE Analysis Between Diploids and Tetraploids Using mRNA-Seq

**FIGURE 3:**
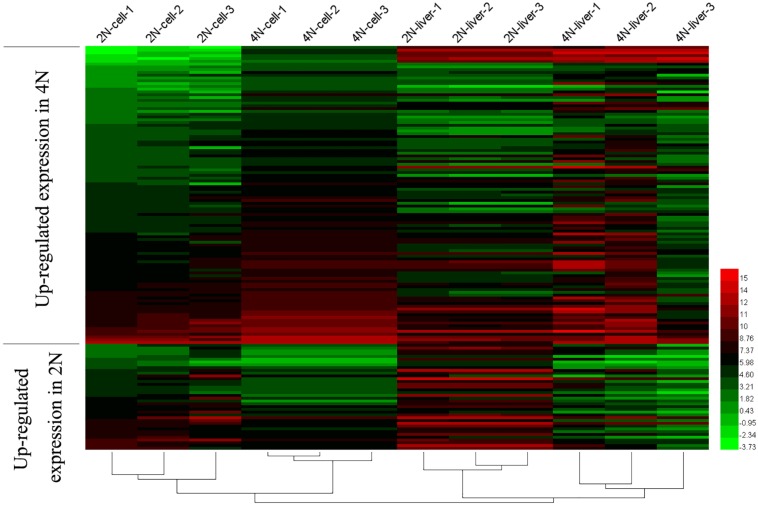
Shared DEGs between diploids and tetraploids in cultured cells and liver tissues. The 145 shared DEGs, including 38 that were upregulated in 2N and 107 that were upregulated in 4N, were detected from the comparison between diploid and tetraploid states.

A comparison of diploids and tetraploids can provide insights into the regulatory mechanisms associated with different ploidy levels. Therefore, we focused on DE analysis between diploid and tetraploid cultured cells of 20,771 genes (vs. 1 in Figure 1A), and found that 19,238 (92.6%) were not DEGs while 1,532 (7.4%) were DEGs; these included 747 (3.6%) that were upregulated in diploid cultured cells and 784 (3.8%) that were upregulated in tetraploid ones (Figure 2A). A comparison of diploid and tetraploid liver samples (vs. 2 in Figure 1A) showed that 486 (2.3%) genes were upregulated in diploid liver tissues, while 1166 (5.6%) were upregulated in tetraploid ones (Figure 2B). In total, 19,238 (92.6%) and 19,048 (92.1%) genes exhibited no significant DE in cultured cells (vs. 1 in Figure 1A) and liver tissues (vs. 2 in Figure 1A), respectively. Of the 20,771 total shared expressed genes, 18,050 (87.0%), including 17,905 (86.2%) non-DEGs and 145 (0.7%) DEGs, were found to have the same expression trend in the comparisons of “vs. 1” and “vs. 2” in Figure 1A (Figure 2C). Of these 145 DEGs, 38 (0.2%) showed upregulated expression in a diploid state, while 107 (0.5%) were upregulated in a tetraploid state (Figures 2D,E). Additionally, the 145 shared DEGs were displayed in a heatmap, in which diploid and tetraploid liver tissue and cultured cell samples were clustered together (Figure 3).

### DEGs Related to the Cell Cycle Pathway

**FIGURE 4:**
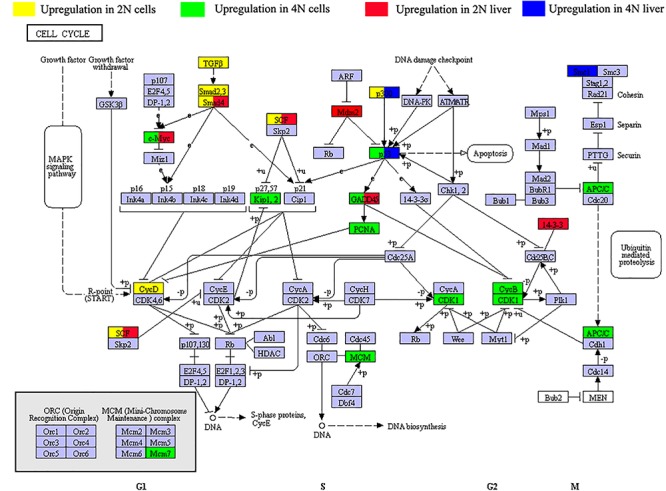
Distribution of differentially expressed genes (DEGs) of the cell cycle pathway (dre04110) in diploid and tetraploid states *in vitro* and *in vivo*. Green symbols represent upregulated expression in 2N cultured cells, yellow symbols represent upregulated expression in 4N cultured cells, red symbols represent upregulated expression in 2N liver, and blue symbols represent upregulated expression in 4N liver. Three genes (*ep300a*, *myc*, and *gadd45*) show the same DE trends between diploids and tetraploids. However, three other genes (*smad4a*, *cul1a*, and *tp53*) exhibited the opposite DE trends.

To investigate changes in cell cycle regulation *in vitro* and *in vivo*, we next focused on KEGG pathways of the DEGs in our result (Supplementary Table 3). In comparison of diploid and tetraploid liver samples (vs. 1 and 2 in Figure 1A), the DEGs were shown to be mainly involved in the ribosome pathway (ko03010, 67 DEGs) and pathways associated with cancer (ko05200, 51 DEGs). Among these, 11 and 21 DEGs were associated with the cell cycle, respectively (Figure 4). Comparing of diploid and tetraploid cultured cells identified 15 DEGs in the cell cycle pathway. Of the seven DEGs shared between diploids and tetraploids in cultured cells and liver tissues, four showed the same expression trends as genes of the cell cycle pathway (Figure 4). Interestingly, three genes (*ep300a*, *myc*, and *gadd45*) exhibited the same DE trends between diploids and tetraploids, while three genes (*smad4a*, *cul1a*, and *tp53*) showed the opposite DE trends.

### Expression Level Determination Using qPCR

**FIGURE 5:**
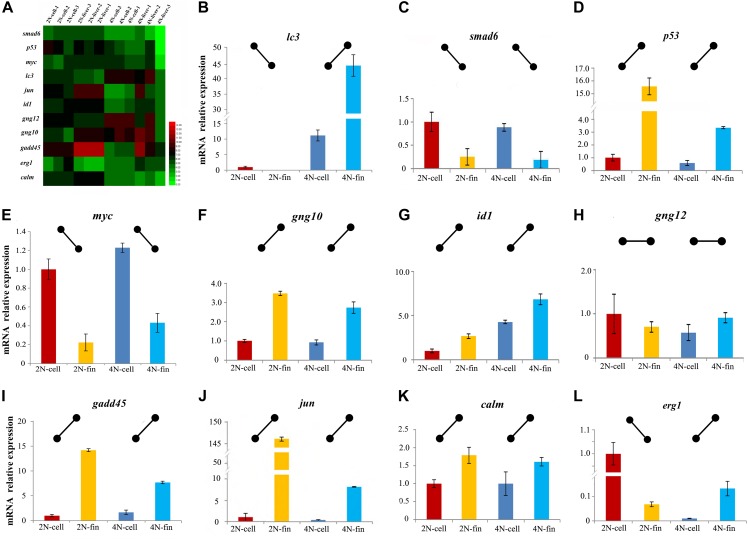
Expression levels of 11 genes detected by mRNA-seq and qPCR. **(A)** Heatmap of the expression distribution of 11 as detected by mRNA-seq in cultured cells and liver tissues. **(B–L)** The expression levels of 11 genes detected by qPCR in cultured cells and caudal fin.

To better investigate expression differences *in vitro* and *in vivo* (Figure 5A), 11 DEGs including those in cell cycle pathways were analyzed with qPCR. This was performed in cultured cells and liver tissues, as well as in fin tissue from which the cultured cells had been generated. The different conditions between cultured cells and tissues resulted in major differences in expression profiles. To better describe gene regulation in the four samples, we established expression patterns based on relative levels in cultured cells and caudal fin tissue (Figures 5B–L). These patterns provided a clear perspective to assess differences between diploids and tetraploids *in vitro* and *in vivo*. The same relative expression patterns between diploids and tetraploids were detected in nine genes (*smad6*, *p53*, *myc*, *id1*, *jun, gng10*, *gng12*, *gadd45*, and *calm*), while different relative expression patterns were detected for the two other genes (*lc3* and *erg1*) (Figure 5). These results of *in vitro* and *in vivo* exhibited the common trend of gene expression in cell-cycle-regulated genes accompanied with tetraploid formation.

## Discussion

Polyploidy were always observed in plant, but rarely in animals (Soltis et al., 2003). The formation of allotetraploid hybrids of *C. auratus* red var. and *C. carpio* L. provided an effective animal model to investigate mechanisms of polyploidy in animal (Liu et al., 2001, 2016). In comparison of diploid and tetraploid individuals, appropriate cell line were urgently needed to discover the different traits related to growth, fertility and disease resistance and various changes in molecular mechanisms for studying the potential mechanisms of these differences (Liu et al., 2001, 2009; Long et al., 2009; Ren et al., 2016). Here, we assessed the diploid and tetraploid cultured cell in gene expression level, and discussed them whether could be used to study polyploidy as comparison to *in vitro*.

Genome-wide expression profiles of polyploid culture cells and tissues in the present study provided a novel insight into the molecular mechanisms underlying the polyploidization effect *in vitro* and *in vivo*. To evaluate expression profile similarities between diploid and tetraploid states *in vitro* and *in vivo*, we performed DE analysis using mRNA-seq and qPCR. The analysis identified many DEGs between cells and liver tissues, not just in the diploid state but also in tetraploids (vs. 3 and 4 in Figure 1A) (Figure 2), indicating that marked changes in mRNA expression may be related to factors including changes in the cell microenvironment and the origin of the material (Arkhipchuk and Garanko, 2005). However, in the comparison between diploid and tetraploid samples (vs. 1 and 2 in Figure 1A), similar expression trends, including 38 shared upregulated genes in diploids, 107 shared upregulated genes in tetraploids, and 17,905 shared genes with no DE, were found *in vitro* and *in vivo* (Figures 2D,E). The results preliminary suggested that the relatively stable expression trends be maintained in most genes irrespective of *in vivo* and *in vitro*.

Dramatic mRNA expression changes often occurred with hybridization and polyploidization (Leggatt and Iwama, 2003; Osborn et al., 2003; Mallet, 2007). Some DEGs distributed were observed in some aquatic organisms, including oysters (Marie et al., 2006), protogynous wrasse (Jeong et al., 2009), rice field eel (Huang et al., 2005), rainbow trout (Cleveland and Weber, 2014), and gibel carp (Sun et al., 2010; Li et al., 2014). However, gene expression of polyploid cultured cell was rarely reported. Focused on cell-cycle-regulated genes, which play an important role in cell proliferation, ontogenesis and survival (Nishihara, 1997; Ashcroft and Vousden, 2001; Boxer and Dang, 2001; Ruzinova and Benezra, 2003; Wimmer et al., 2010; Wisdom et al., 2014; Valente et al., 2015), the 11 genes had been selected and performed with expression analysis using qPCR. The same expression trends were detected in nine genes between cultured cells from fin and caudal fin tissues (Figure 5), further suggesting that the common trends of gene expression were in cell-cycle-regulation irrespective of *in vivo* and *in vitro*. This research focused on common characteristics between diploids and tetraploids, providing us the gene expression changes of polyploidization *in vitro* and *in vivo*. Our findings indicate that the cultured cell line of this study appears to be an appropriate platform for polyploidy research, especially into the regulation of cell proliferation and adaptive regulation, although further comparisons of diploid and tetraploid material are necessary.

## Data Availability Statement

RNA-Seq data were submitted to NCBI SRA (SRR7640867, SRR7640866, SRR7640869, and SRR7640868).

## Ethics Statement

All experiments were approved by the Animal Care Committee of Hunan Normal University and followed guidelines of the Administration of Affairs Concerning Animal Experimentation of China. All samples are raised in natural ponds, dissections are performed under sodium pentobarbital anesthesia, and all efforts are made to minimize suffering. This manuscript does not involve the use of any human data or tissue.

## Author Contributions

LR, SL, JiamL, and YX wrote and modified the manuscript. LR, JiahL, YF, and YH provided assistance extracting the raw material, and performing the qPCR experiment and bioinformatics analyses. YX and SL contributed to the conception and design of the study. All authors read and approved the final manuscript.

## Conflict of Interest

The authors declare that the research was conducted in the absence of any commercial or financial relationships that could be construed as a potential conflict of interest.

## Footnotes

1 http://www.bioinformatics.babraham.ac.uk/projects/fastqc/, version 0.11.3

2 https://www.ncbi.nlm.nih.gov/genome/?term=goldfish

3 http://rd.biocloud.org.cn/
