# Supplementary material for: Expression Profile Analysis of the Cell Cycle in Diploid and Tetraploid Carassius auratus red var
Source: Front Genet. 2020 Mar 17;11:203. doi: 10.3389/fgene.2020.00203 (PMC7089929; doi:10.3389/fgene.2020.00203)
Supplement: Supplementary file 1 [file Table_1.pdf]

Supplementary Table 1. The summary of qPCR primer.

	Forward Primer	Reverse Primer
p53	AAGGGTAAGGAAAGATACGAG	CAGCATACCCTTCTTTCCA
gadd45	GTCGTTGGATGCAATATCAC	CAGTACGACACAATCTGG
myc	GATTACGACTACGACTCCTACC	AAACTCCGACACCATTTCC
jun	GGCTCATCATCCAGTCCA	CTGTTGTTGATTGTTGTTTGGG
calm	CGAGTTTCTGACAATGATGG	CGTAATTGACCTGACCGT
id1	TCTCCAGGACATGAACAG	CTGATTCTTCTTGCTATCCAG
smad6	ATTCATCAAGTCAGCCACAC	GAATATGACCTCCAGCCAG
lc3	GGAATCTTCCGAATGAGCACAG	CTTCCTGATGATCCACATGAAC
gng12	CTGAGAGTCGAGGCCAGTATT	GTAACGCATGAGGTCAGCT
gng10	CACGACCATGAAGCGCAC	TTGATTCTGTCCACGCTG
erg1	GATGATCATGCTGAACTCTG	CAGACTGTTACTGCAGTTATG
