# Supplementary material for: Expression Profile Analysis of the Cell Cycle in Diploid and Tetraploid Carassius auratus red var
Source: Front Genet. 2020 Mar 17;11:203. doi: 10.3389/fgene.2020.00203 (PMC7089929; doi:10.3389/fgene.2020.00203)
Supplement: Supplementary file 2 [file Table_2.pdf]

Supplementary Table 2. The summary of mapping to reference genome.

Sample	NO. clean reads (million)	NO. mapped reads (million)	Mapped ratio
	37.7	25.9	68.65%
2n-cell	43.8	29.8	68.05%
	51.2	45.6	88.88%
	41.2	31.6	76.75%
4n-cell	42.7	32.7	76.68%
	41.9	31.6	75.44%
	54.6	50.9	93.13%
2n-liver	40.0	37.3	93.14%
	35.2	32.1	91.30%
	71.8	65.9	91.83%
4n-liver	27.7	25.7	92.75%
	48.1	42.6	88.55%
