# Supplementary material for: Expression Profile Analysis of the Cell Cycle in Diploid and Tetraploid Carassius auratus red var
Source: Front Genet. 2020 Mar 17;11:203. doi: 10.3389/fgene.2020.00203 (PMC7089929; doi:10.3389/fgene.2020.00203)
Supplement: Supplementary file 4 [file Presentation_1.pdf]

Expression Profile Analysis of the Cell Cycle in Diploid and Tetraploid *Carassius auratus*

Li Ren[1,2†], Jiahao Lu[1,2†], Yunpeng Fan[1,2], Yibo Hu[1,2], Jiaming Li[1,2], Shaojun Liu[1,2*], and Yamei Xiao[1,2*]

[1]State Key Laboratory of Developmental Biology of Freshwater Fish, Hunan Normal University, Changsha, 410081, Hunan, P.R. China

[2]College of Life Sciences, Hunan Normal University, Changsha, 410081, Hunan, P.R. China

[†]These authors contributed equally to this work.

[*]Authors for correspondence:

Prof. Yamei Xiao: yameix@hunnu.edu.cn
State Key Laboratory of Developmental Biology of Freshwater Fish, Hunan Normal University
Changsha 410081, China
Tel.: 86-731-88873074

Prof. Shaojun Liu: lsj@hunnu.edu.cn
State Key Laboratory of Developmental Biology of Freshwater Fish, Hunan Normal University
Changsha 410081, China
Tel.: 86-731-88873074

Supplementary Figure

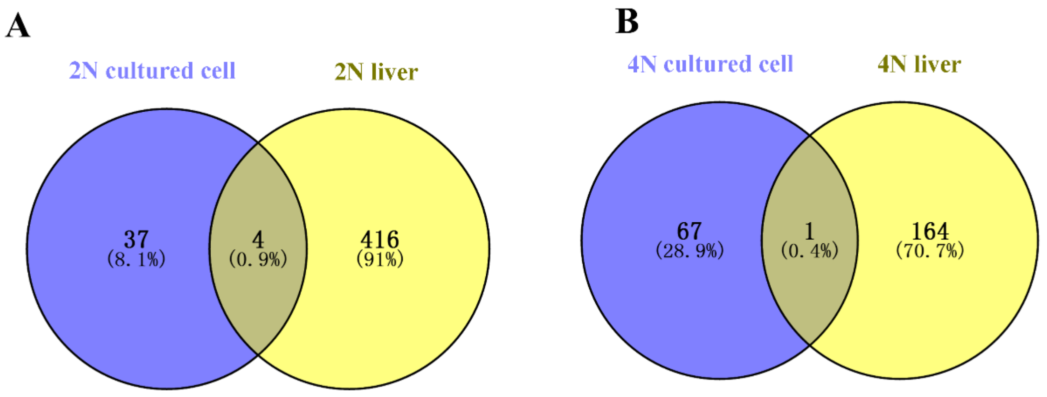

Supplementary Fig. 1. Silent transcripts in the comparison of diploid and tetraploid detected by mRNA-Seq. A. The distribution of silent transcripts detected in diploid cells and liver. B. The distribution of silent transcripts detected in tetraploid cells and liver.

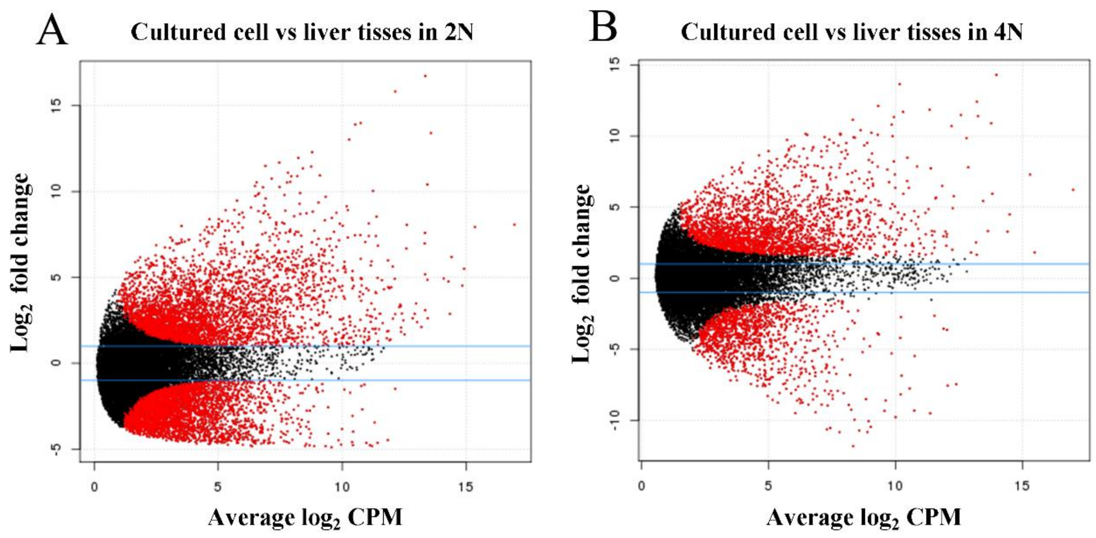

Supplementary Fig. 2. Heatmap of DE transcripts between cultured cell and liver tissues. A. The distribution of DE transcripts between diploid cultured cells and liver tissues. B. The distribution of DE transcripts between tetraploid cultured cells and liver tissues. Log_2 counts per million (CPM).

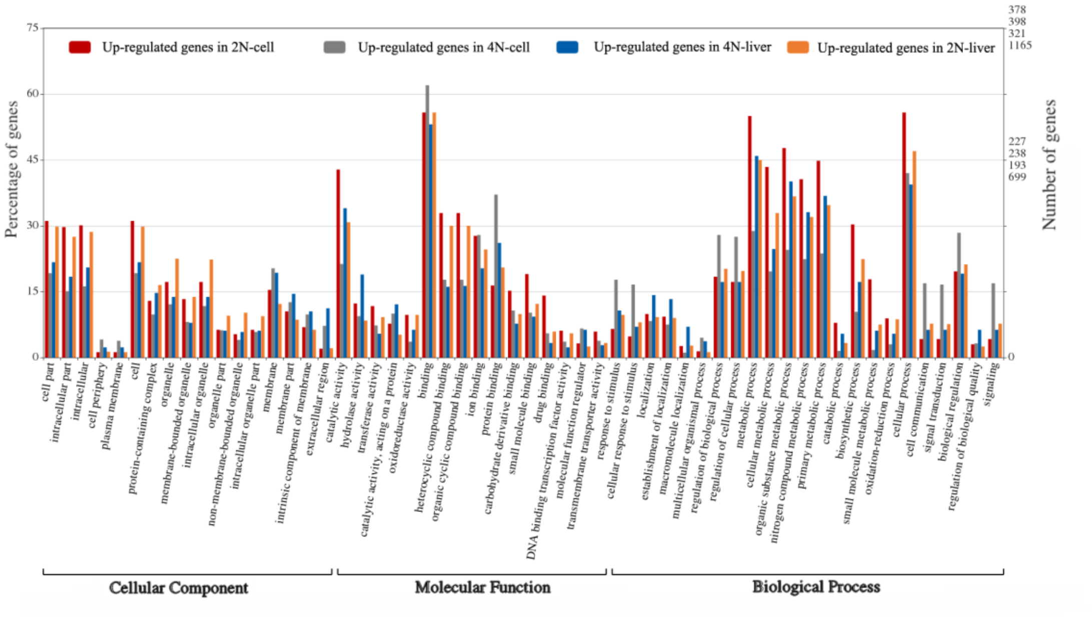

Supplementary Fig. 3. Gene Ontology (GO) assignments for the DE transcripts between diploid and tetraploid in vivo and in vitro. GO assignments (level 2) were used to predict the distribution of functional genes in transcriptome data. The findings for three categories are shown: cellular component, molecular function, and biological process.
